# Surgical treatment of rare peripheral nerve lesions: long-term outcomes and quality of life

**DOI:** 10.3389/fonc.2024.1476019

**Published:** 2025-02-26

**Authors:** Andrija Savić, Milan Lepić, Jovan Grujić, Aleksa Mićić, Aleksandra Stojiljković, Gunna Hutomo Putra, Andrej Terzić, Lazar Vujić, Lukas Rasulić

**Affiliations:** ^1^ Faculty of Medicine, University of Belgrade, Belgrade, Serbia; ^2^ Clinic for Neurosurgery, University Clinical Centre of Serbia, Belgrade, Serbia; ^3^ Faculty of Medicine, University of Defence, Belgrade, Serbia; ^4^ Clinic for Neurosurgery, Military Medical Academy, Belgrade, Serbia; ^5^ Department of Neurosurgery, Rumah Sakit Universitas Airlangga, Surabaya, Indonesia

**Keywords:** rare diseases, peripheral nerves, peripheral nerve tumors, neurosurgery, patient outcome assessment, quality of life

## Abstract

**Introduction:**

Rare peripheral nerve lesions comprise a histologically diverse group of neoplastic and non-neoplastic entities, characterized by infrequent occurrence and variable clinical presentations, presenting significant diagnostic and therapeutic challenges. This study presents eight cases of surgically treated rare peripheral nerve lesions with previously unreported long-term outcomes involving quality of life (QOL) assessment.

**Methods:**

A retrospective analysis was conducted on medical records from 2012 to 2022 to identify surgically treated cases of rare peripheral nerve lesions, selecting eight cases based on determined inclusion and exclusion criteria. Long-term outcomes and QOL were assessed 12 months post-surgery by patient examination, control imaging and self-reporting questionnaires.

**Results:**

The study included 4 benign (hemangioblastoma, angiomatoid fibrous histiocytoma, endometriosis (n=2)) and 4 malignant lesions (NTRK-rearranged spindle cell neoplasm, lymphoma, metastatic breast carcinoma (n=2)). Even though benign lesions generally presented with better outcomes, this was more closely related with level of nerve invasion and postoperative sequele, rather than presence of malignancy.

**Discussion:**

Because of a global lack of experience in handling such cases, this study aimed to present the cases we encountered in detail to serve as a basis for future literature reviews. The findings highlight the importance of individualized treatment strategies and long-term follow-up to optimize functional recovery and patient well-being.

## 1 Introduction

Rare medical diseases lack an internationally accepted consensus on their definition. They are usually defined by having a prevalence of less than 0.04%, with specific thresholds differing according to each country’s standards (1–5). According to these criteria, diseases of the peripheral nervous system are relatively common, with prevalence estimates reaching up to 24%, depending on the study inclusion criteria (6–14).

However, no specific definitions or prevalence data exist to classify peripheral nerve diseases as rare. According to some authors, excluding the most common etiologies reveals a variety of histopathological entities that could be considered rare due to their low or unreported individual occurrence (15, 16). According to others, all infrequently encountered, rarely reported, and poorly studied peripheral nerve lesions could be considered rare. The surgically treated cases mostly include benign or malignant neoplastic (tumors), as well as non-neoplastic (tumor-like) lesions, which may originate from neural (primary/intrinsic/neurogenic) or surrounding (secondary/extrinsic/non-neurogenic) tissue (17–21).

Primary peripheral nerve tumors are not rare in surgical practice, accounting for up to 12% of all benign and 8% of all malignant soft-tissue tumors (22). They mostly include benign peripheral nerve sheath tumors (PNSTs), such as schwannomas and neurofibromas, which are not considered rare according to their prevalence rates (17–19, 23). All remaining benign PNTSs listed in the WHO classification list, such as peri-neurinoma, granular cell tumor, malignant peripheral nerve sheath tumor (MPNST), hybrid PNST, and unusual variants of schwannomas and neurofibromas are considered rare (20). Despite being considered rare, some of these lesions are sufficiently studied and reported in the literature, such as MPNST.

The MPNSTs are extremely rare, with an estimated prevalence of 0.001% within the general population (24). However, owing to their relatively higher occurrence within neurofibromatosis, they are not so rarely encountered, comprising up to 10% of all soft-tissue sarcomas and 10% of all surgically treated PNSTs (25–27). However, MPNST is a heterogeneous group of pathological entities, and some forms such as epithelioid are even rarer. In addition, regarding genomic heterogeneity in soft-tissue sarcomas, some genomic MPNST variants are also extremely rare or yet unreported in the literature (28).

Secondary peripheral nerve tumors comprise a group of various benign and malignant neoplasms with the potential to infiltrate neural tissue. Non-infiltrative compressive lesions should not be considered peripheral nerve tumors. All secondary peripheral nerve tumors are rare and, upon infiltration of neural tissue, become non-neural sheath tumors (18).

The malignant secondary nerve lesions usually affect the brachial and lumbal plexuses, with prevalence rates in cancer patients up to 0.43% and 0.71%, respectively (29). Because of anatomical proximity, up to 4.9% of breast carcinoma cases may infiltrate the brachial plexus, being the most reported and well-studied type of metastatic peripheral nerve disease. The lesser but significant amount includes brachial plexopathy induced by lung carcinoma. The remaining types of metastatic nerve involvement comprise a large group of individually rare presentations, mostly underreported and poorly studied in the literature (43).

Benign secondary peripheral nerve tumors are a large group of heterogeneous histological entities with low individual occurrence, often misconceived with tumor-like lesions. The tumor-like lesions are also a heterogeneous group of individually rare entities occurring due to various etiologies. Throughout the literature, some of these entities were transferred from one category to another, while others were continuously discussed in the same way. For example, ganglion nerve cysts are the most frequent tumor-like lesions, sometimes presented as neoplastic lesions in the literature. Nevertheless, they were considered rare in the past and are now being increasingly diagnosed and reported due to more frequent usage of nerve imaging modalities (17–20, 22).

Owing to their infrequent occurrence and diverse clinical presentation, rare peripheral nerve lesions present diagnostic and treatment challenges. The limited available literature often provides diverse and incomparable study results, leaving surgeons to rely heavily on personal experience in managing these cases. This paper aims to evaluate long-term outcomes and quality of life (QOL) in eight patients who underwent surgical treatment for rare peripheral nerve lesions. By providing new insights, this study seeks to enrich the existing literature and offer a reference point for future research (20, 40, 44, 50).

## 2 Materials and methods

A retrospective analysis of patients’ medical records was performed at the author’s department for 10 years (1 January 2012 to 31 December 2022) to select the surgically treated cases of rare peripheral nerve lesions. The eight selected cases were included in the study according to the following inclusion and exclusion criteria.

### 2.1 Inclusion criteria

- Surgically treated patients for rare peripheral nerve lesions during the selected period
- Electromyoneurography (EMNG), ultrasound (US), and magnetic resonance imaging (MRI) verified nerve lesion
- Intraoperatively verified infiltration of the nerve
- Performed biopsy and histopathological analysis
- The pathohistological diagnosis is
       ◦ noted in less than five cases during the selected period or
       ◦ not findable as a case report in the literature or
       ◦ already described in the literature as a rare peripheral nerve lesion or
       ◦ with unreported incidence and prevalence rates, or
       ◦ mostly reported through individual case reports
- Regular follow-up minimum 1 year after the surgery
- Signed patient approval to participate in the study

### 2.2 Exclusion criteria

- Surgically treated patients for cranial or spinal nerve pathology
- Preoperative disability due to other nerve injury
- The pathohistological diagnosis is
       ◦ noted in more than five cases for a selected period (ganglion nerve cyst and MPNST)
       ◦ intraoperatively excluded infiltration of the nerve (epithelioid sarcoma)
       ◦ already described in the literature as a non-rare lesion (ganglion nerve cyst)

### 2.3 Outcome assessment

The postoperative evaluation was performed 1 day, 14 days, and 12 months after surgery. The outcome after 12 months was considered as long-term. On the first day, the patient examination included an assessment of pain and a Color Doppler Scan to exclude vein thrombosis. On the 14th day after surgery, the examination included pain and motor strength assessment and stitch removal. QOL was assessed 12 months after surgery.

- Pain was assessed using Visual Analog Scale (VAS) for pain.
- Motor strength was examined by the Medical Research Council (MRC) Manual Muscle Testing (MMT).
- QOL was assessed using SF-36 (Short form 36) and PNSQOL (Peripheral Nerve Surgery Quality of Life) questionnaires (**Supplementary Material 1**).

### 2.4 Data description and writing

The manuscript was prepared based on a thorough analysis of the collected data. The manuscript was drafted and refined with the assistance of ChatGPT (version January 2025, GPT-4-turbo, OpenAI) to improve clarity and coherence. The final manuscript was critically reviewed and edited by the author to ensure accuracy, scientific integrity, and compliance with journal guidelines.

## 3 Results

**Figure 1:**
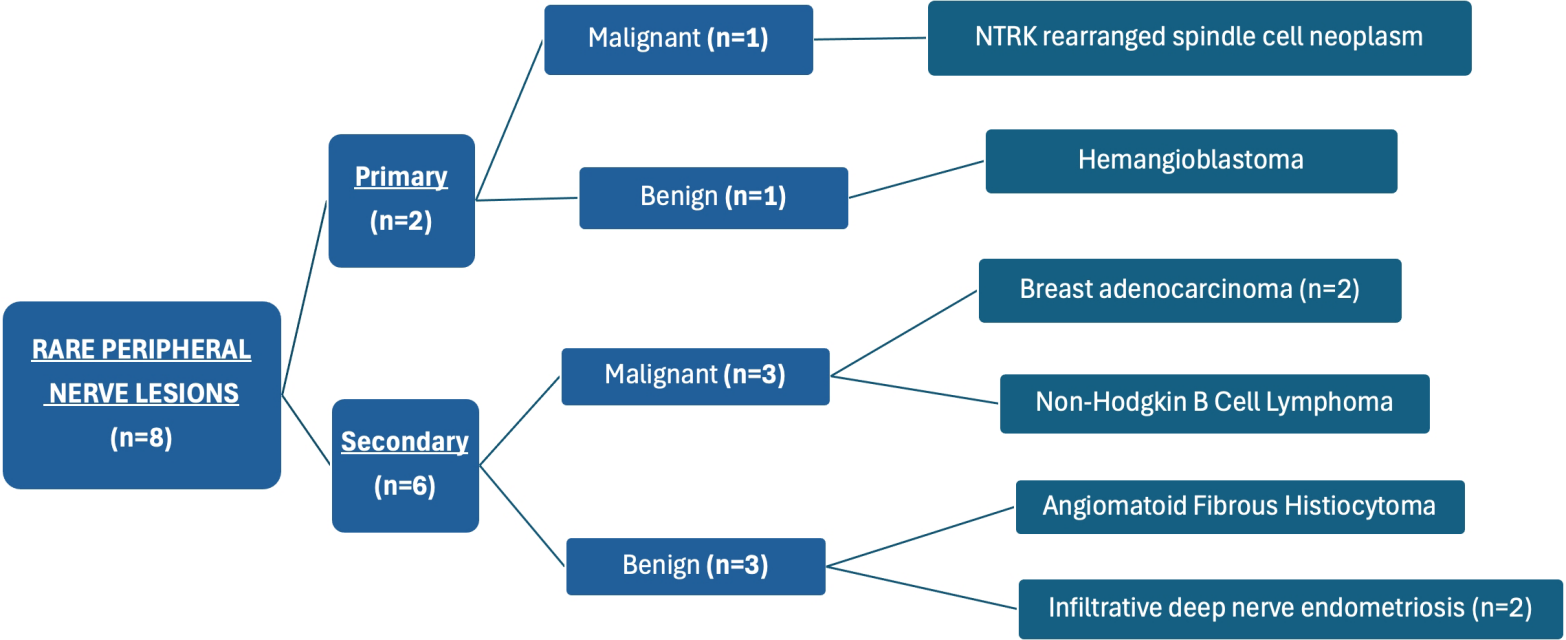
Distribution of the study cases according to their characteristics.

Our case series included two primary lesions, originating from the neural tissue, and six secondary lesions, originating from surrounding tissue (**Figure 1**). There was an equal amount of benign and malignant lesions (4:4). Two cases—ulnar nerve hemangioblastoma and isolated sciatic nerve endometriosis—have already been reported in the literature by some of the authors from our team but without long-term outcomes and QOL assessment (30, 31).

### 3.1 NTRK rearranged spindle cell neoplasm of the tibial nerve

A 51-year-old man presented with an expansive mass in the right popliteal fossa. He initially noticed the mass 2 years earlier, with gradual enlargement, which became more pronounced over the last 6 months. Prior to admission, he had undergone two unsuccessful needle aspirations of the popliteal mass, because a regional medical center doctor had mistakenly identified it as a Becker’s cyst.

#### 3.1.1 Clinical evaluation

**Figure 2:**
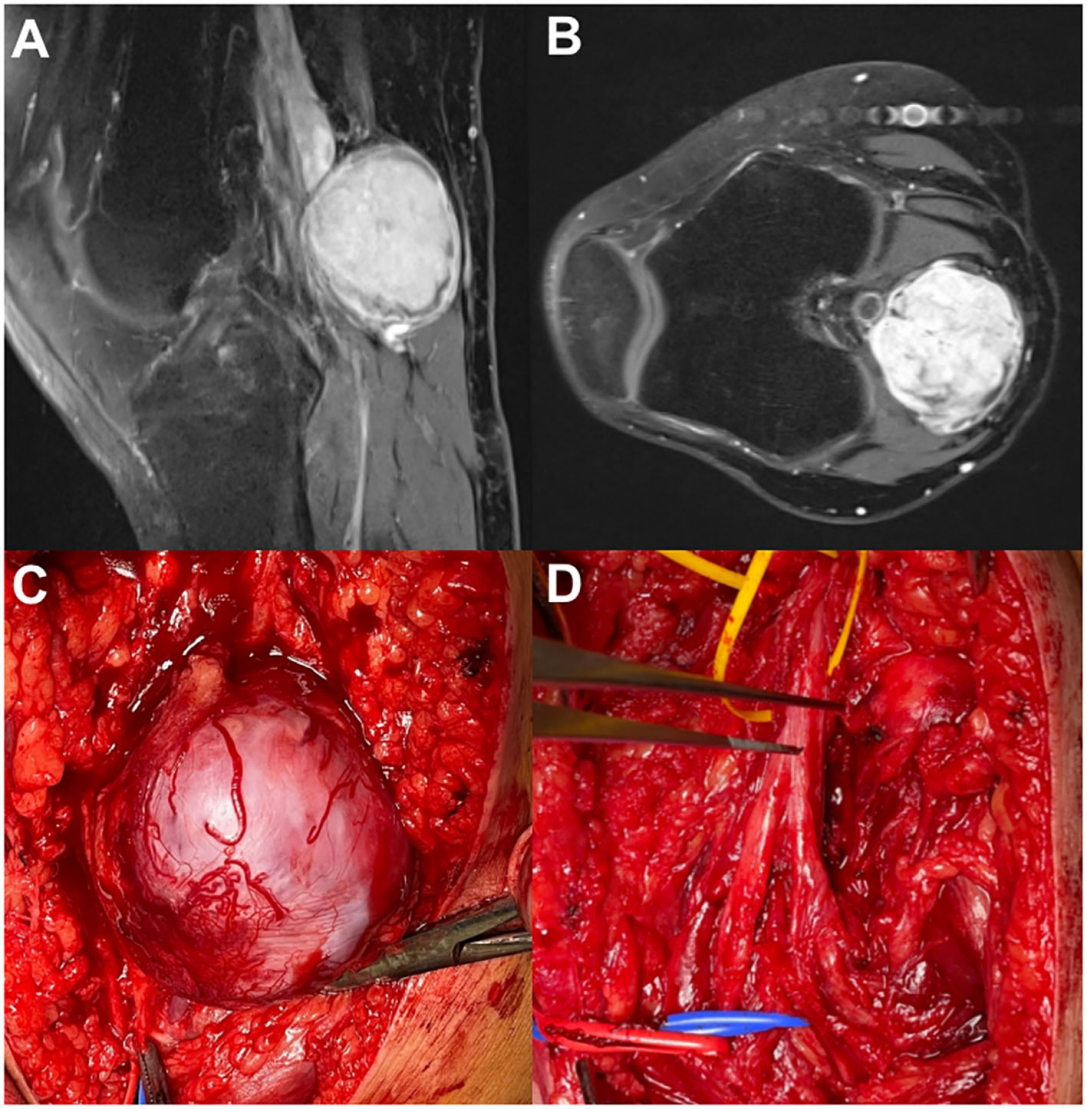
NTRK rearranged spindle cell neoplasm. **(A)** MRI—sagittal view. **(B)** MRI—axial view. **(C)** Open exploration (popliteal approach) of the tumor and tibial nerve. **(D)** Tibial nerve after tumor removal.

Physical examination revealed a demarcated expansive mass approximately 3 cm in diameter palpable in the medial part of the right popliteal fossa. Neurologic examination excluded motor deficits, while the patient reported cyclic occurrence of pain radiating from the knee to the lower back (VAS = 3). Both USG and MRI of the right knee confirmed the presence of a tumor in the popliteal fossa, clearly demarcated from surrounding structures and morphologically suggestive of a tibial nerve schwannoma (**Figure 2**).

#### 3.1.2 Surgical procedure

The patient was placed in a prone position, and the right tibial nerve was explored using a medial popliteal approach. The encapsulated tumor, sized up to 5 cm, originated from the tibial nerve. The tumor capsule was promptly vascularized and adherent with the vessels from the surrounding tissues. Upon dissection of the tumor capsule, identification, and transection of the originating fascicles, the sole tumor removal was not possible due to its fibrotic adherence to a large popliteal blood vessel. An attempt to separate the tumor from the underlying large vein resulted in vein rupture, requiring immediate vascular repair.

The tumor was covered with a grayish-white membrane, measuring 53 × 44 × 43 mm. On cross-section, the tissue had a soft-elastic to tough consistency, heterogeneous appearance, grayish-white, and light brown color, structureless, fibrous, and partly nodular, with irregular yellowish reticular zones and solid, compact areas of firmer consistency, with no clear signs of necrosis or bleeding.

#### 3.1.3 Short-term follow-up

On the first day after surgery, there were no changes in the overall status of the patient. Considering performed vascular repair, the patient was hospitalized for a few days longer than planned and daily checked for deep vein thrombosis using CDS. On the fifth day after surgery, the thrombosis was verified and the patient was examined by a vascular surgeon and then discharged with appropriate advice and medicamentous therapy. Two weeks after surgery, there were no changes in the patient’s general and neurologic status. He was examined by a vascular surgeon and radiologist and advised for further self-management.

Histopathological, immunohistochemical, and FISH analyses proved the diagnosis of NTRK rearranged spindle cell neoplasm, with an NTRK1 gene present in approximately 20% of analyzed nuclei. According to established regulations, the patient was admitted to the Council for Soft-Tissue Tumors of Extremities, which serves as a referral body in our country. The Council indicated a CT scan of the body to exclude metastatic dissemination. Upon exclusion, he was advised to be actively involved in regular imaging controls to prevent potential progressive tumor recurrence.

#### 3.1.4 Long-term follow-up

There were no significant changes in the long-term functionality of this patient. Twelve months after our surgery, he still receives therapy for deep vein thrombosis and lives satisfied, knowing that there are no signs of tumor dissemination or local recurrence, relying on regular CT and MRI controls. At examination, the patient presented with local pain in the right knee (VAS = 3), which was not associated with the tumor surgery, but rather a recent injury considering MRI findings of a bone bruise in the medial tibial condyle, post-traumatic changes in the posterior medial collateral ligament, and swelling of the medial collateral ligament. Despite that, his SF-36 scores indicate strong physical functioning at 0.85, with no significant limitations in physical or emotional roles, both scoring 1.0. Energy is high at 0.8, and emotional wellbeing and social functioning are positive at 0.8 and 1.0, respectively. The PNSQOL score was almost maximal (77/80). The patient has only mild difficulties in recreational activities and no challenges with work tasks. There are minimal issues with pain and sleep, showing that discomfort may be well-managed. Socially and emotionally, experiences of pity are rare, with no reported discrimination, and the tumor has not impacted social life or daily activities. Satisfaction is very high in all areas, including the condition of the extremity, social life, and professional life, indicating that the patient experiences minimal limitations and maintains wellbeing across physical, social, and emotional domains.

### 3.2 Ulnar nerve hemangioblastoma

A 70-year-old man presented with a slow-growing, palpable mass on his right upper arm, present for 2 years. His main complaint was discomfort during palpation and consequential propagation of paresthesia into the right hand.

#### 3.2.1 Clinical evaluation

Physical examination located the palpable Tinel-positive mass at the anterior side of the right upper arm. Neurologic examination excluded motor weakness. An EMNG test was conducted, revealing an ulnar nerve lesion in the right upper arm associated with bilateral median nerve entrapment at the level of carpal tunnel.

#### 3.2.2 Surgical procedure

**Figure 3:**
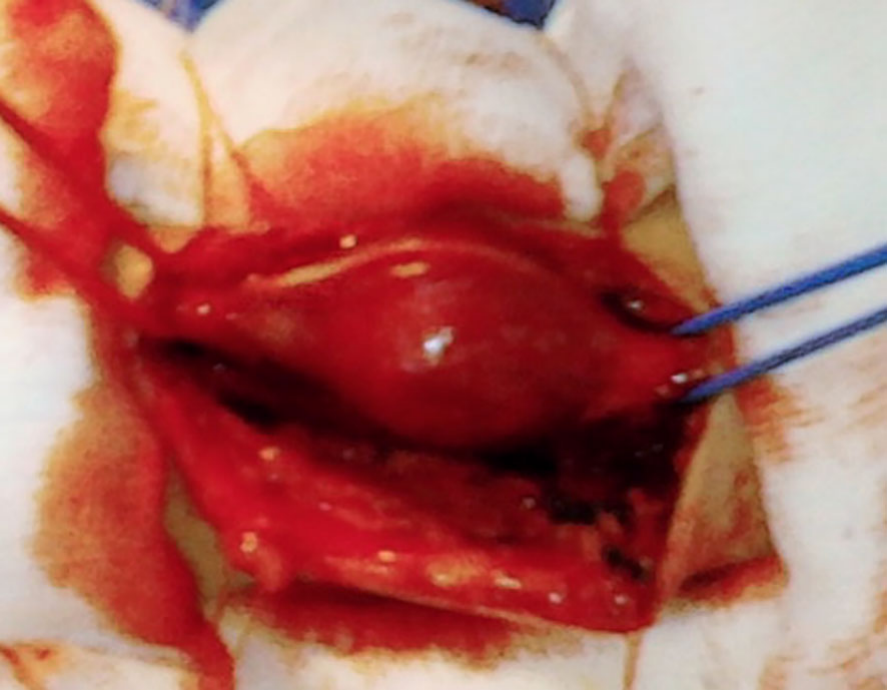
Ulnar nerve hemangioblastoma. Reposted courtesy of Prof. Lukas Rasulić.

The patient was placed in a supine position with an abducted upper arm and an extended forearm. The tumor was round, encapsulated, reddish-orange, well-vascularized, up to 2.5 cm in diameter, with one nerve fascicle entering its tissue (**Figure 3**). All fascicles were carefully dissected and remained intact.

#### 3.2.3 Short-term follow-up

On the first day after surgery, there were no changes in the neurological findings compared to before surgery. After 2 weeks, the patient reported a reduction in paresthesia intensity and occurrence. Histopathological and immunohistochemical analysis verified a peripheral nerve hemangioblastoma, WHO grade I. The patient was referred for physical therapy.

#### 3.2.4 Long-term follow-up

Twelve months after surgery, the patient presented with a complete absence of paresthesia radiating from the upper arm to the hand. The only impairment that remained was hypoesthesia of the fourth and fifth fingers. However, his QOL data show generally positive outcomes. Physical functioning is high at 0.85, indicating minimal limitations and role limitations are moderate, with scores of 0.75 for physical and 0.78 for emotional aspects. Energy is robust at 0.9, while emotional wellbeing at 0.64 and social functioning at 0.75 suggest stable emotional health and good social interactions. According to PNS QOL scores (76/80), the patient experiences mild difficulties in recreational and work activities, reports no sleep issues, and has not experienced feelings of pity or discrimination. Satisfaction levels are high, as well as social and professional life, reflecting well-maintained QOL across all areas.

### 3.3 Brachial plexus neurolymphomatosis

A 14-year-old female patient presented with right shoulder pain and progressive weakness of the right arm during the last 7 months. Because of the patient’s claims that the onset of symptomatology was associated with a quick, inappropriate shoulder move that she made, most physicians considered it traumatic and referred her to physical therapy. There were no signs of recovery after 6 months of physical therapy.

#### 3.3.1 Clinical evaluation

On admission, a neurologic examination revealed complete right brachial plexus palsy (MRC 0). EMNG indicated a suspectable lesion of posterior and lateral brachial plexus fascicles, with possible involvement of superior and middle trunks. MRI indicated an increased T2 signal of the C5, C6, and C7 roots and their distal branching with suspectable chronic epi- and intraneural hematomas.

#### 3.3.2 Surgical intervention

**Figure 4:**
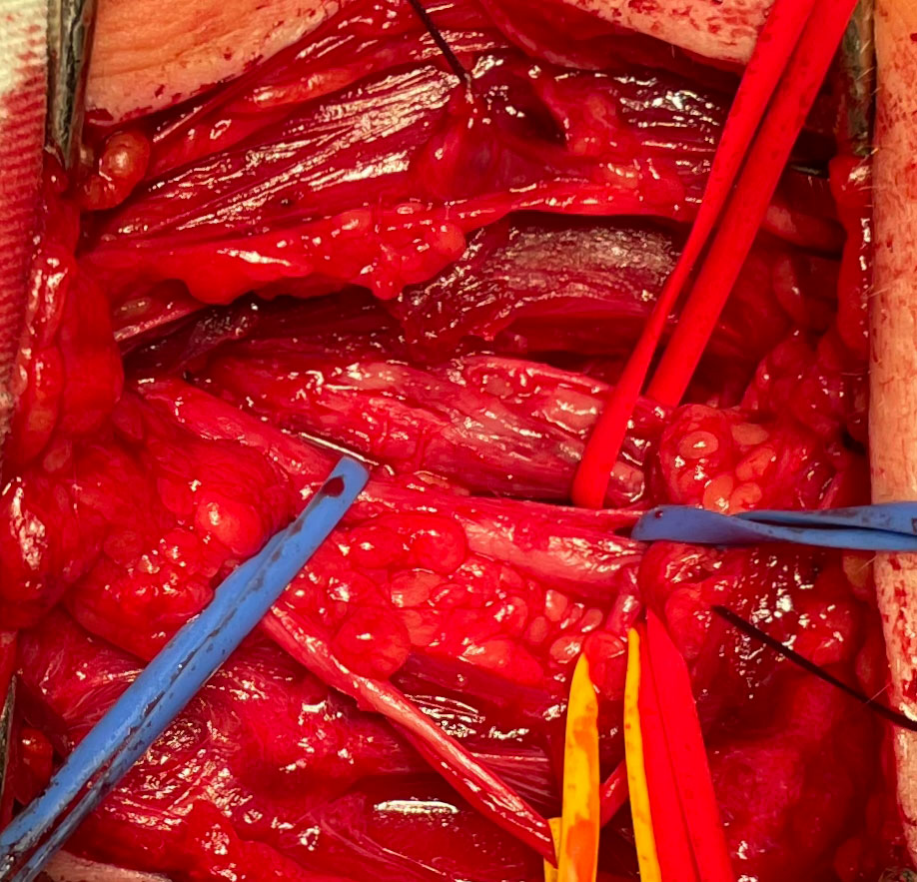
Brachial plexus neurolymphomatosis (supraclavicular exploration).

The patient was placed supine, and supraclavicular brachial plexus exploration was performed. During the surgery, superior and middle brachial plexus trunks were sclerotic and neoplastically altered, fused, and compressed by the surrounding tissue (**Figure 4**). Surgical decompression and external neurolysis were performed, followed by electrodiagnostic testing. Since there was no signal conduction during direct nerve stimulation, an incision biopsy of the altered nerve elements was performed.

#### 3.3.3 Short-term follow-up

There were no signs of recovery up to 1 month after surgery. Histopathological and immunohistochemical findings indicated a large B-cell non-Hodgkin lymphoma. The FISH analysis showed the C-MYC and BCL-2 gene rearrangement, indicating a definitive diagnosis of a “double-hit” high-grade B cell lymphoma (centroblast, non-GCB type). The patient was referred to a pediatric hematologist for evaluation and further management.

Six months after surgery, a progression of disease was noted, presented by the development of left leg weakness (MRC 3). An MRI indicated an extensive neoplastic dissemination affecting most body parts. The patient was then subjected to various combinations of chemotherapeutic agents, complicated by allergic reactions to many of them (rituximab, methotrexate, bactrim, and pentamidine). After three cycles of therapy, leg strength started to recover, and after six cycles, the arm started to recover. After the seventh cycle, MR findings indicated regression of disseminated disease.

#### 3.3.4 Long-term follow-up

Twelve months after surgery, the patient achieved significant functional recovery in terms of regaining muscle strength of the leg (MRC 5), elbow flexion (MRC 5), and shoulder abduction (MRC 3). The PET/CT finding verified that there are no active focuses of the disease.

In this case, the SF-36 scores reveal an excellent QOL with minimal limitations. Physical functioning is nearly optimal at 0.95, and there are no physical or emotional role limitations, both scoring 1.0. High energy (0.9), strong emotional wellbeing (0.92), and no pain (1.0) indicate physical comfort and stable mental health. Social functioning is unaffected (1.0), with the patient experiencing no sleep issues, pity, or discrimination.

PNSQOL scores (53/80) confirm full independence in daily and professional activities, and satisfaction levels are very high across all areas, including social and professional life. Overall, the patient experiences minimal impact from the lymphoma, maintaining an active, independent life with strong wellbeing across physical, social, and emotional dimensions.

### 3.4 Infraclavicular neoplastic brachial plexopathy due to breast carcinoma

A 46-year-old woman with a history of right mastectomy (6 years earlier) and chemotherapy due to breast carcinoma presented with pain and progressive weakness of the right arm during the last 2 years. Neurologic examination showed a painful (VAS = 10) extended right upper brachial plexus palsy (MRC 1-2). EMNG and MRI evaluation indicated an infiltrative lesion affecting the infraclavicular portions of the medial and posterior fascicles.

**Figure 5:**
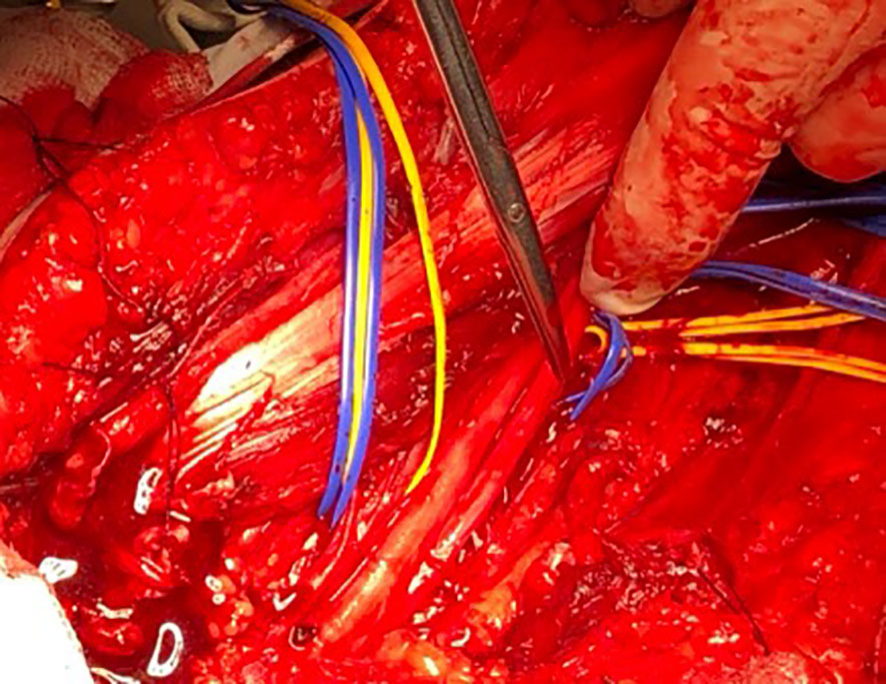
Infraclavicular neoplastic brachial plexopathy due to breast carcinoma (infraclavicular exploration).

The patient was placed in a supine position, and an infraclavicular approach was used to explore the plexus. Intraoperative findings included scarring tissue compressing the brachial plexus elements, with an expansive infiltrative lesion of the medial and posterior fascicles (**Figure 5**). Decompression and external neurolysis were performed, followed by incision biopsy.

The patient was satisfied with the surgery after the first day due to significant pain relief (VAS = 5). Two weeks after surgery, the pain almost completely diminished (VAS = 2), but there was no improvement in the arm strength. The histomorphological analysis showed the recurrence of the previously treated breast carcinoma. The patient was referred to an oncologist and biological therapy was started.

Twelve months after surgery, the patient reports improvement in QOL in terms of the absence of pain, while there was no functional recovery. However, her SF-36 and PNSQOL results indicate a reduced QOL across multiple domains.

The SF-36 scores show moderate physical functioning at 0.5, with severe limitations in both physical and emotional roles, each scoring 0, suggesting significant challenges in daily responsibilities and emotional strain due to physical limitations. Energy is low at 0.3, reflecting persistent fatigue, while emotional wellbeing is also low at 0.32, indicating a considerable impact on mental health. Social functioning is minimal at 0.125, possibly related to physical discomfort as indicated by a pain score of 0.525, and the overall perception of general health is poor at 0.2.

PNSQOL (47/100) results further highlight challenges with independence and social interactions, with moderate difficulty reported for personal hygiene and severe limitations in household tasks and recreational activities, indicating significant restrictions in daily life. The patient often experiences pity and occasional humiliation, which may exacerbate emotional challenges and contribute to social isolation. Despite these difficulties, the patient reports moderate satisfaction with social life and high satisfaction in their professional life, possibly reflecting coping mechanisms or support in structured environments. The combined SF-36 and PNSQOL results suggest that while the patient faces considerable physical and social limitations, they show resilience in maintaining aspects of social and professional satisfaction.

### 3.5 Supra-infraclavicular neoplastic brachial plexopathy due to breast carcinoma

A 57-year-old woman with a history of partial resection of breast carcinoma presented with right cervico-brachial syndrome and arm weakness. She complained of pain (VAS = 10) for the last 3 years, and multiple attempts of physical therapy modalities were attempted without signs of improvement. After some time, the patient experiences almost complete paresis of the right arm.

MRI of the cervical spine showed a massive expansive formation in close relation with adjacent blood vessels, bone, and nerves on the right side. Neurological examination revealed complete brachial plexus palsy with hypotrophy of all muscles. The tumor was palpable in both the supraclavicular and infraclavicular regions.

An infraclavicular decompression with biopsy was performed. There was immediate pain relief (VAS = 4) following surgery. Post-operative histopathological findings showed a morphological finding that corresponds to adenocarcinoma metastasis.

The SF-36 scores of this patient indicated substantial physical limitations (0.4) and severe restrictions in both physical and emotional roles, scoring 0 for each. This patient faces chronic fatigue (energy score of 0.3) and lower emotional wellbeing (0.36), reflecting both physical and mental challenges. Social functioning is low (0.125), and general health is also poor (0.2). PNSQOL (44/80) scores reveal severe difficulty with personal care and household tasks, hindering independence. The patient experiences occasional pity and rare discrimination, indicating moderate resilience. However, professional satisfaction is low, in contrast to moderate satisfaction in social life, reflecting the strain on regular functioning due to physical limitations.

### 3.6 Isolated sciatic nerve endometriosis

A 45-year-old woman presented with cyclic occurrence of sciatica in the right leg for the last 3 years with a progression during the last year. Before our examination, the patient was conservatively managed in other institutions and considered a case of degenerative lumbosacral disease.

#### 3.6.1 Clinical evaluation

Upon admission, refractory sciatic pain (VAS = 9) was the patient’s main complaint, associated with reduced motor strength of all muscle groups of the right leg (MRC grade 4). EMNG was used to confirm sciatic nerve injury. An MRI showed a non-demarcated expansive lesion, up to 4 cm in diameter, located in the piriformis muscle lodge, infiltrating the muscle and sciatic nerve (**Figure 4**).

#### 3.6.2 Surgical procedure

**Figure 6:**
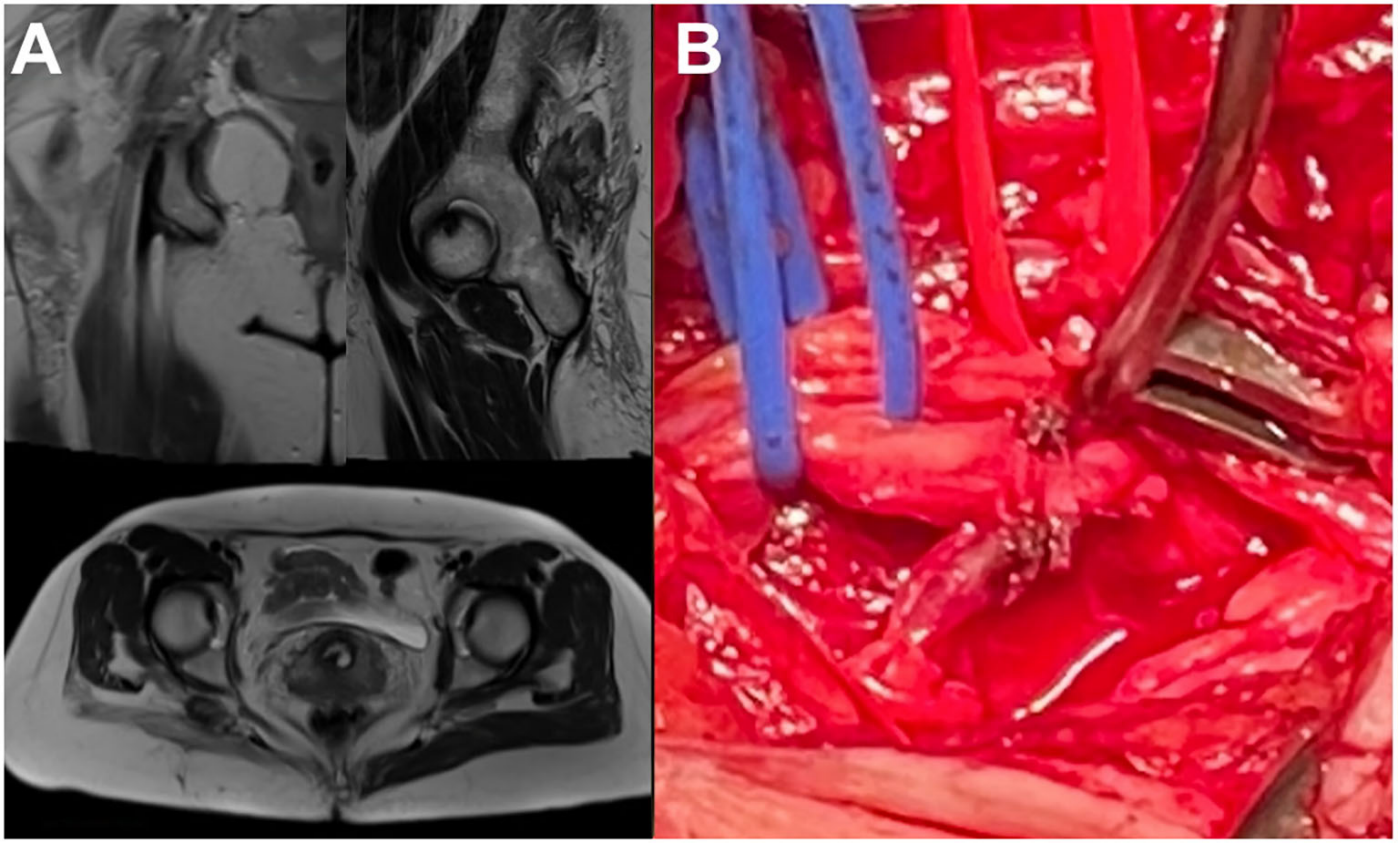
Isolated sciatic nerve endometriosis. **(A)** MRI finding. **(B)** Intraoperative finding (transgluteal approach).

The patient was placed in a prone position and the sciatic nerve was explored through an open transgluteal approach (**Figure 6**). A macroscopic finding included a diffusely altered sciatic nerve in the piriform canal, compressed by calcified fibrous tissue. Microscopic findings revealed a diffusely changed and thickened sciatic nerve, filled with dark liquid cysts. Surgical decompression, release, and external neurolysis were performed, followed by an incision biopsy of a cyst and surrounding altered nerve tissue.

#### 3.6.3 Short-term follow-up

On the first postoperative day, the patient was satisfied and reported pain relief (VAS = 6) while muscle weakness of the left leg progressed [foot flexion (MRC grade 3) and extension (MRC grade 2)].

Histomorphological findings indicated a deep infiltrative sciatic nerve endometriosis. The patient was referred to physical therapy and a gynecologist for evaluation and further management, who prescribed triptorelin therapy for 6 months. During the therapy, the patient experienced significant pain relief (VAS = 3) and recovery of leg strength [foot flexion (MRC grade 5) and extension (MRC grade 3)]. She was able to walk independently without supporting devices.

Triptorelin was excluded from the therapy after 6 months, followed by progression of pain and gait disturbances. The patient was referred to a neurosurgeon for an opinion on the necessity of repeating the therapy concerning the patient’s QOL. The neurologic exam revealed the weakness of the foot flexion (MRC grade 3) and extension (MRC grade 1), associated with intensive sciatic pain (VAS = 8) and being unable to walk without supporting devices. The patient was referred back to the gynecologist with advice to repeat triptorelin therapy.

#### 3.6.4 Long-term follow-up

Upon continuation of the gynecologic medical treatment, the patient recovered in terms of pain reduction (VAS = 2) but maintained weakness of foot extension (MRC grade 2). SF-36 scores show moderate physical limitations (0.4) but strong emotional resilience, with an emotional role score of 1.0. The patient experiences some fatigue (energy score of 0.3) but maintains stable emotional wellbeing (0.64) and excellent social functioning (1.0). Minimal pain (0.9) suggests physical discomfort is limited, allowing independence in daily tasks per PNSQOL (68/80) findings. Social and professional satisfaction is moderate, indicating a balanced QOL.

### 3.7 Associated sciatic nerve endometriosis

A 40-year-old woman presented with pain and paresthesia in the lower back during the last 2 years, being more severe during the menstrual cycle. The last 5 months were characterized by progression in terms of paresthesia propagation into all fingers of the right foot, altering her gait performance. Three years earlier, the patient was subjected to intrapelvic endometriosis surgery.

#### 3.7.1 Clinical evaluation

**Figure 7:**
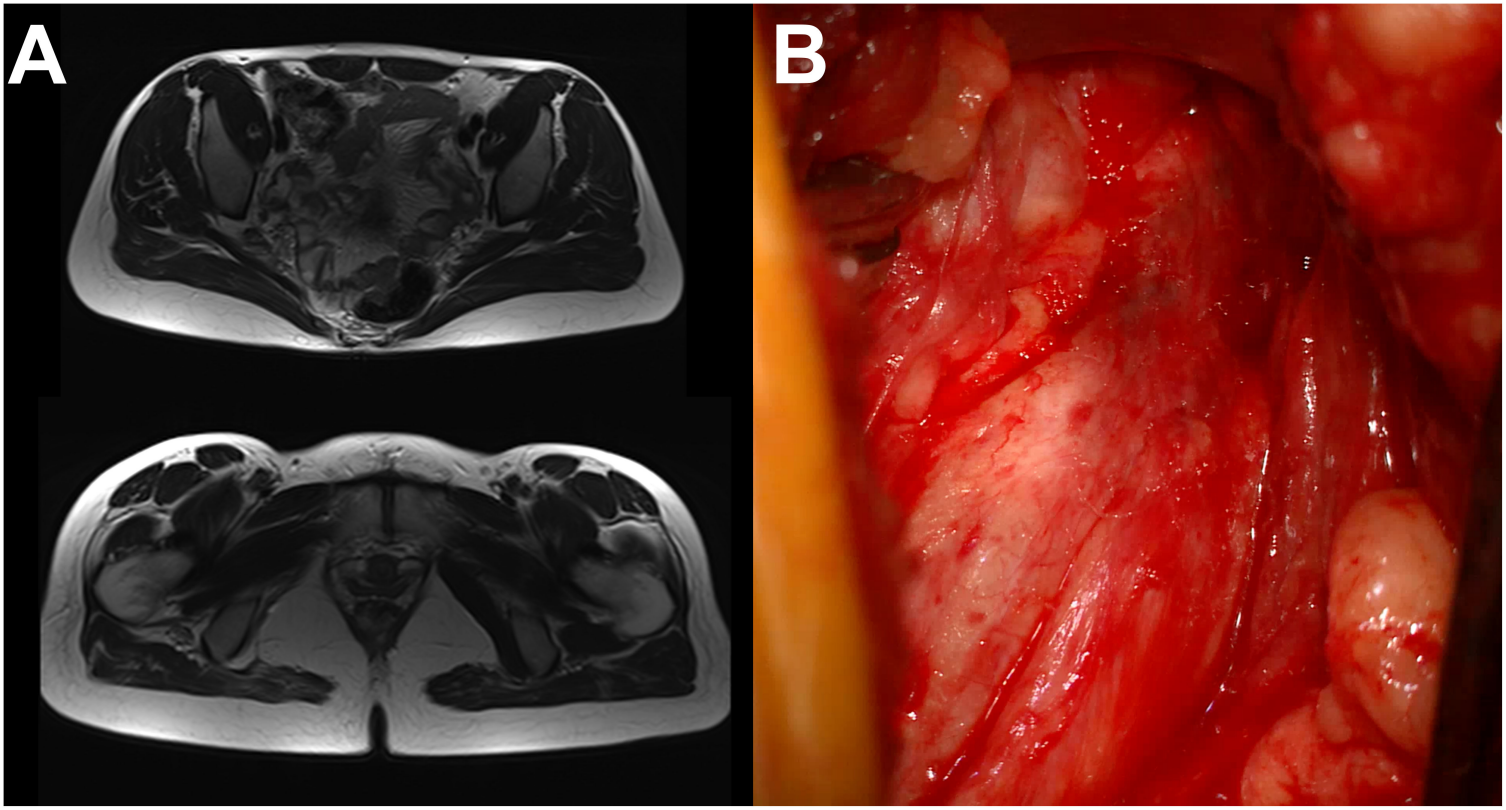
Sciatic nerve endometriosis. **(A)** MRI finding. **(B)** Intraoperative microscopic finding (transgluteal approach).

Upon admission, the patient’s main complaint was propagating right leg paresthesia ending with foot hypoesthesia and consequential disbalance-associated difficulties in walking. The motor strength of all muscle groups of the right leg was reduced (MRC grade 3), while the foot exhibited deformities with contractures. The lower back pain (VAS = 6) that occasionally accompanied the paresthesias was not significant, according to the patient’s claims. Upon EMNG verification of the sciatic nerve lesion, the USG indicated a preserved nerve continuity associated with piriform and gemellus muscle tendinitis. An MRI revealed a non-homogeneous thickening of the right sciatic nerve at the level of the ischio-femoral space (**Figure 7**).

#### 3.7.2 Surgical procedure

The patient was placed in a prone position, and the right sciatic nerve was explored through an open transgluteal approach. In both macroscopic and microscopic findings, the sciatic nerve was sclerotic and adherent to surrounding tissue. A careful decompression and external neurolysis were performed, followed by an incision biopsy of epineurium infiltrated by adherent sclerotic tissue.

#### 3.7.3 Short-term follow-up

The first day after surgery, the patient reported a slight reduction of paresthesia without evident improvement in gait performance. Two weeks after surgery, the muscle strength of the right leg improved (MRC 4) with evident progress in walking function. One month after surgery, the patient presented with complete muscle strength recovery (MRC 5), absence of pain, and paresthesias while only complaining about the foot deformity.

Histomorphological and immunohistochemical findings revealed micro-focuses of endometriotic tissue infiltrating the epineurium. The patient was referred to a gynecologist for further management. However, the gynecologist assumed that the endometriotic tissue was removed from the sciatic nerve and, upon USG exclusion of intrapelvic endometriotic involvement, decided not to prescribe hormonal therapy.

Six months after surgery, the patient experienced symptom exacerbation, presented as intermittent leg pain (VAS = 9), muscle weakness (MRC 3), and walking inability. An MRI revealed progression in size of the endometriotic tissue affecting the right sciatic nerve. The patient was readmitted to a gynecologist to reconsider hormonal therapy. One month later, upon the patient’s request, she was subjected to a bilateral oophorectomy.

#### 3.7.4 Long-term follow-up

Twelve months after our surgery and 5 months after the bilateral oophorectomy, the patient experienced incomplete muscle strength recovery (MRC grade 4), reduction of pain (VAS = 3), and paresthesia while lacking improvement in foot deformity. SF-36 scores reveal moderate physical functioning (0.65) and balanced scores in energy (0.6) and emotional wellbeing (0.72), indicating overall resilience. Social functioning and general health are moderately high (0.75), pointing to a positive outlook. PNSQOL results (68/80) indicate mostly independent daily functioning, with occasional difficulty in specific tasks. Experiences of pity are infrequent and do not appear to affect the patient significantly. Satisfaction scores are moderate across social and professional domains, suggesting that the patient has adapted well to the condition despite some physical restrictions. During examination, she stated that she is satisfied with her current QOL and is looking forward to achieving a complete functional recovery.

### 3.8 Angiomatoid fibrous histiocytoma of the saphenous nerve

A 33-year-old woman presented with pain and paresthesias in the left popliteal fossa propagating through the anterior side of the lower leg during the last 3 years. The patient’s main complaint was extremely intense, sharp, propagating pain (VAS = 10) initiated by even the slightest touch.

#### 3.8.1 Clinical evaluation

Muscle strength was preserved in all muscle groups. There were no gait disturbances. There was no pain in the resting state. EMNG findings confirmed a saphenous nerve lesion, while USG revealed a hypoechogenic expansive lesion involving the nerve. The MRI finding characteristics of the lesion suspected a neurofibroma. The lesion was below skin level and was not visible by inspection, and palpation revealed its location by inducing the pain with minimal pressure.

#### 3.8.2 Surgical procedure

**Figure 8:**
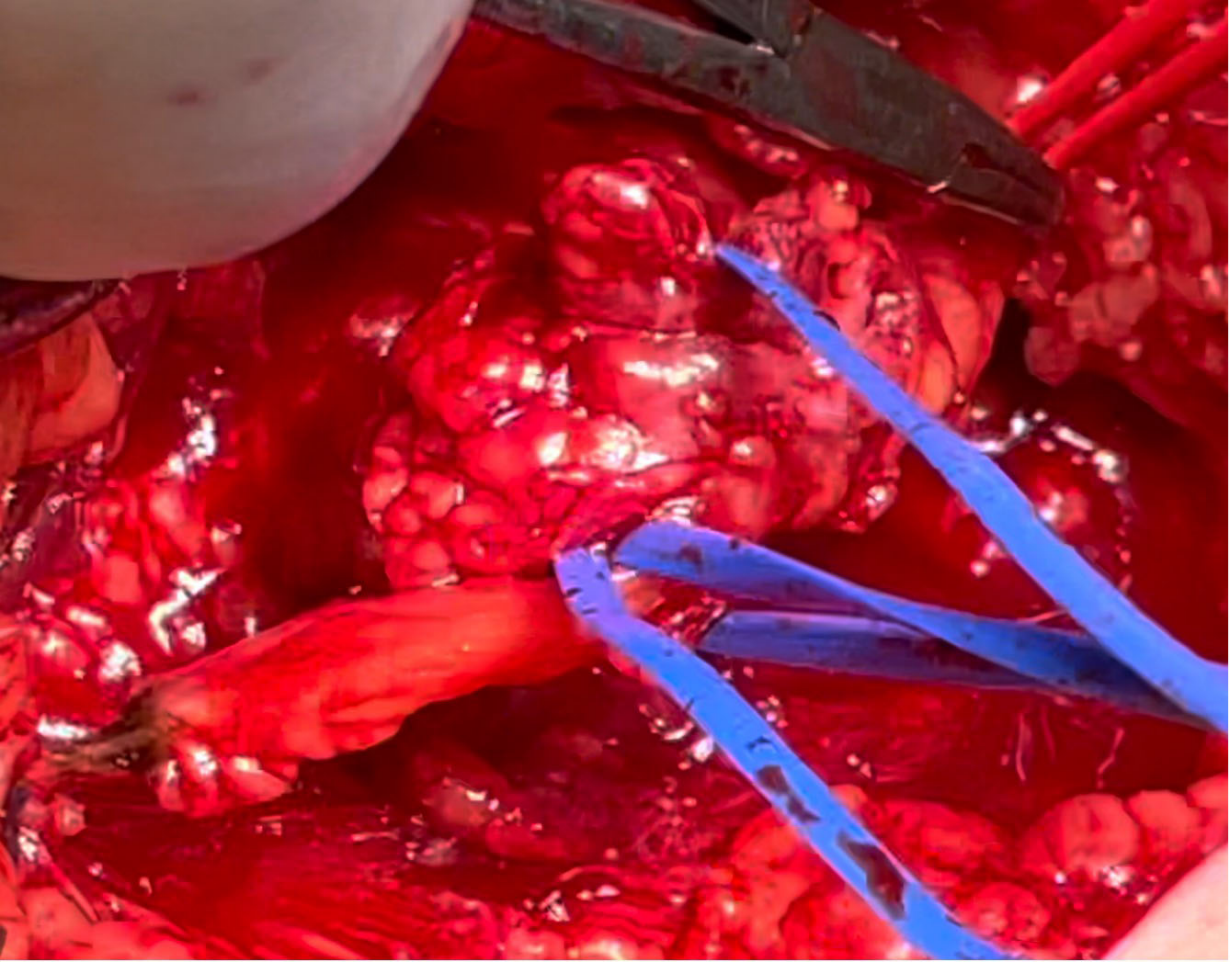
Intraoperative macroscopic finding of saphenous nerve AFG (popliteal approach).

The patient was placed in the lateral position, providing a medial approach to the popliteal fossa. The intraoperative finding revealed a tumor of fibrous consistency and very adherent to surrounding tissues involving the entire diameter of the saphenous nerve (**Figure 8**). The tumor was dissected from surrounding tissues and removed by transecting the proximal and distal parts of the saphenous nerve. The following act included debridement of the surrounding soft tissues covered with adhesive neoplastic tissue.

#### 3.8.3 Short-term follow-up

On the first postoperative day, the muscle motor strength was preserved, while intensive preoperative pain could not be induced by palpating the skin over the triggering projection. The patient only complained about the pain around the operative wound (VAS = 2), which was limiting her walking.

Two weeks after surgery, there was no pain (VAS = 0) and paresthesia, leading to complete functional recovery. Histomorphological, immunohistochemical, and FISH analyses indicated a mesenchymal tumor with the characteristics of angiomatoid fibrous histiocytoma (AFH). According to established regulations, the patient was admitted to the Council for Soft-Tissue Tumors of Extremities, which serves as a referral body in our country.

Two months after surgery, the patient remained asymptomatic. The CT scan of the body was used to exclude malignant dissemination. However, a follow-up MRI revealed a local tumor recurrence without involving the transected nerve stumps. Despite the patient having no complaints, she was submitted to an indicated revision surgery performed by an orthopedic surgeon. Such revision surgery aims to prevent another local recurrence by providing gross total tumor resection and more radical debridement of surrounding tissues.

#### 3.8.4 Long-term follow-up

Twelve months after our surgery, the patient presented with loco-regional knee pain (VAS = 4) and lower leg lymphedema, persisting for the past 10 months as a consequence of revision surgery. In addition, the most recent MRI finding indicated a local re-recurrence of the tumor.

The SF-36 scores revealed moderate physical functioning (0.5) and severe role limitations in both physical and emotional aspects, each scoring 0. Energy and emotional wellbeing scores are moderate at 0.65 and 0.68, respectively, reflecting some resilience despite fatigue. Social functioning and pain are also moderate (0.5 and 0.45), suggesting occasional discomfort that might affect social interactions. PNSQOL (52/80) findings indicate that the patient is independent in most daily activities but struggles with household chores. Occasional pity and rare humiliation suggest mild emotional challenges, though satisfaction remains moderate in social and professional life.

## 4 Discussion

Rare peripheral nerve lesions individually represent an extremely small portion of all nerve cases but display various presentations and, collectively, become more impactful. Because of a global lack of experience in handling such cases, this study aimed to present the cases we encountered in detail to serve as a basis for future literature reviews. The following text discusses each study case individually, as well as within its histological category, and in comparison with all other cases, focusing on factors that reduce or enhance QOL.

### 4.1 Peripheral nerve NTRK rearranged spindle cell neoplasm

Neurotrophic tyrosine receptor kinase (NTRK) neoplasms are a rare subset of soft-tissue tumors characterized by gene fusions involving NTRK1, NTRK2, or NTRK3. These fusions lead to constitutive activation of TRK proteins, driving oncogenesis. While NTRK fusions are documented across various tumors, their occurrence in spindle cell neoplasms, particularly those originating from nerve tissues, is uncommon. NTRK rearranged spindle cell neoplasms (NTRK-RSCNs) are a rare type of NTRK fusion-positive sarcomas that share some diagnostic and treatment challenges with other soft-tissue sarcomas while differing in the need for specialized molecular testing, as well as variable clinical presentation and nature of surgical complications (32–34).

NTRK-RSCNs can present as a slow-growing mass and therefore be misdiagnosed as benign tumors, which initially occurred in our patient. Their expansive growth is usually characterized by adhesive attachment to surrounding structures, which occurred in our patient, and led to a vascular nerve lesion requiring immediate surgical repair. On the other side, these NTRK-RSCNs can present as a fast-growing mass, which can be misdiagnosed with MPNSTs.

Timely recognition of NTRK-RSCNs is crucial to avoid misdiagnosis as benign lesions, given their potential for recurrence and dissemination. Conversely, they should not be mistaken for MPNST, as NTRK-RSCNs have a lower recurrence rate and very rarely disseminate. This distinction typically means that surgery alone is sufficient without adjuvant therapy, though regular imaging follow-ups are necessary with a potential for targeted immunotherapy.

To our knowledge, there are no reports on long-term outcomes and QOL in patients treated for nerve NTRK-RSCNs. We consider that QOL in these patients, as in patients with soft-tissue sarcomas in general, will primarily depend on the extent of nerve and surrounding tissue invasion, the severity of postoperative sequelae, and the recurrence rate. A study on QOL in high-grade soft-tissue sarcomas found that while patients recover well physically from surgery, the mental component showed no improvement, regardless of age, highlighting the need for comprehensive care that addresses both physical and mental health. Since the recurrence and dissemination rates are low in patients with NTRK-RSCNs, mental support may be out of greatest significance. The long-term outcome in our case showed a high QOL with strong physical and social functioning, minimal pain, and high emotional wellbeing—outcomes that align with literature findings on the benefits of proactive care and psychosocial support in maintaining QOL for similar cases.

### 4.2 Peripheral nerve hemangioblastoma

Hemangioblastomas are rare benign vascular tumors that mostly occur in the CNS, with an incidence rate of 0.141 per 100,000 person-years according to one study (35). There are only a few cases in the literature arising from the peripheral nerves usually presented as pain in the innervation field of the affected nerve (18, 30, 36–39). Surgical resection is a primary option, which may require nerve resection and repair depending on the severity of intraneural infiltration.

Based on our knowledge, there is a lack of papers in the literature concerning QOL following peripheral nerve hemangioblastoma surgery. The patient presented in our study had no significant deficits before surgery and the treatment was indicated for diagnostic purposes. His long-term outcomes were characterized by reduced severity of preoperative symptoms and no tumor recurrence. Therefore, we consider that in such cases, long-term outcomes and QOL after surgery mostly depend on the severity of nerve infiltration and outcomes of nerve recovery rather than the tumor’s pathological characteristics.

### 4.3 Neurolymphomatosis

Neurolymphomatosis (NL) is a rare manifestation of non-Hodgkin’s lymphoma (NHL), presented as the involvement of peripheral nerves, occurring in approximately 3% of the cases with NHL. Contrary to the categorization of other peripheral nerve lesions, where NL is always considered secondary, NL itself can be either primary, originating within peripheral nerve tissue, though less common, or secondary, invading from surrounding tissues, which is more typical. In most cases, NL involves diffuse large B-cell NHL, affecting the brachial plexus; however, it remains poorly studied in the literature, especially in pediatric cases (40).

A recent meta-analysis aimed to analyze long-term outcomes and prognostic factors in patients with NL, revealing their shorter median survival compared to cases affecting the central nervous system or vascular elements. Furthermore, it emphasized that NL diagnosis is challenging and that the patient’s age and time elapsed from symptom onset to the treatment are the main prognostic factors (41).

The histomorphological features and location of NL in our case align with the most frequent instances reported in the literature, while remaining extremely rare considering the age group. The initial misdiagnosis as a traumatic injury, leading to delayed appropriate treatment, reflects the diagnostic challenges in patients with NL. Even though the time from symptom onset to diagnosis and treatment was longer in our case, compared to the median in the general NL population, the survival time was longer. This may be attributed to the patient’s age, contributing to its impact as a prognostic factor.

There are reports on QOL following brachial plexus lymphomatosis to be compared. The high QOL scores in our case indicate minimal limitations and strong wellbeing, suggesting that with timely and appropriate treatment, patients can achieve favorable outcomes, as highlighted in the literature.

### 4.4 Neoplastic peripheral neuropathy

Peripheral nerve lesions in patients with malignancy occur in 1.7% to 16% of cases, mostly affecting the cervical, brachial, or lumbar plexus (42). Brachial plexus lesions associated with malignancy mostly result from metastatic infiltration [neoplastic brachial plexopathy (NBP)] or radiation therapy [radiation-induced brachial plexopathy (RBP)]. The literature does not provide a calculated incidence of NBP and RBP among patients with malignancy. Some studies report the prevalence of NBP among cancer patients to be 0.43%, while RBP occurs in 1% of patients who have undergone radiation therapy.

Lung or breast carcinoma metastases are the most common causes of NBP (43, 44). Breast-originating NBP is mostly caused by metastatic spreading through the axillary lymph nodes, infiltrating the infraclavicular nerve elements, while direct invasion or vascular spreading is less common. Progressive pain is usually the first symptom, followed by progressive arm weakness distributed within the C8 and T1 root innervation field. Tumor expansion and invasion of the retroclavicular and supraclavicular nerve elements may involve all brachial plexus roots. The onset of symptoms may be delayed, starting many years after diagnosing the primary tumor site. In our study, both cases followed this pattern, with symptom onset delayed by a few years, starting with progressive pain and followed by arm weakness. In both cases, the infiltration involved infraclavicular nerve elements, with one case showing expansion into the retroclavicular space.

Breast-originating NBP is commonly associated with RBP due to the proximity of the radiation target area to brachial plexus elements. Differential diagnosis can be confirmed through neurologic examination, EMNG, USG, or MRI findings. NBP is usually characterized by severe pain as the predominant complaint, along with USG and MRI confirmation of an expansive lesion. In contrast, RBP is characterized by less severe pain, lymphedema, and involvement of the supraclavicular plexus elements, along with EMNG signs of myokymia and MRI evidence of nerve thickening without any focal mass. Both of our patients had a positive history of radiotherapy but presented with pain as the most significant complaint, and USG and MRI verified the presence of the focal mass lesion. It is worth mentioning that it is not unusual to delay the diagnosis due to the similarity of the symptoms with cervical spine pathology, which was seen in one of our patients (45).

The clinical presentation of our patients is comparable with the literature data. The primary indication for surgery in both cases was pain relief, and postoperatively, both patients reported satisfaction with their treatment outcome, as it provided substantial pain relief. However, both patients presented with lower QOL compared to the population with breast carcinoma in general, most likely due to nerve involvement, resulting in pain and functional deficits (46).

The differences in motor deficits most likely contributed to variations in QOL outcomes between the two cases. The infraclavicular case showed moderate physical functioning with some social interaction challenges and moderate satisfaction across social and professional domains. Despite persistent fatigue and moderate difficulties in daily activities, her satisfaction was relatively high in professional life, likely supported by a structured environment that accommodated her limitations. In contrast, the supra-infraclavicular case presented with lower physical functioning and more significant dependency in daily tasks. This greater physical limitation affected her professional satisfaction, which was very low, while she maintained only moderate satisfaction in her social life.

### 4.5 Peripheral nerve endometriosis

Endometriosis is a benign chronic inflammatory disease in which endometrium-like tissue infiltrates the structures outside the uterus, affecting up to 10%–15% of female patients in the reproductive period, with a prevalence rate of up to 2% and annual incidence rates of up to 0.3%, respectively (47, 48). The endometriosis may significantly alter QOL presenting as dysmenorrhea, chronic pelvic pain, right iliac fossa pain, dysuria, dyspareunia, or infertility (49).

In cases affecting the sciatic nerve, the symptoms include cyclic sciatica usually misleading the diagnostic process toward degenerative spine diseases (50). The cases of intrapelvic sciatic nerve involvement that require surgery are usually managed using the laparoscopic approach by the gynecologists (51, 52). In cases of extrapelvic endometriosis with the involvement of the sciatic nerve, an open transgluteal approach may be necessary to diagnose or relieve the symptoms (53). Based on our knowledge, there were no reports on QOL following sciatic nerve endometriosis cases that were treated by an open transgluteal approach (54).

Our study included two cases of extrapelvic sciatic nerve endometriosis, one of which was isolated and the second was associated with intrapelvic involvement. Both cases presented with sciatica and cyclical progression of symptoms, which were reduced immediately following the surgical decompression.

The case with isolated sciatic nerve involvement presented a diagnostic challenge due to the isolated extrapelvic involvement of the sciatic nerve, which was rarely reported in the literature (55). The intraoperative finding was unspecific and unfamiliar with the previous experience, and an attempt to widely explore the nerve and provide a proper biopsy resulted in sciatic nerve injury and consequential leg weakness. In the other case, a positive history of endometriosis treatment and MRI-verified presence of intrapelvic endometriotic tissue played a great role in planning the surgery. A careful non-extensive decompression and biopsy were performed, without postoperative complications and significant improvement of the patient’s functionality.

Comparing QOL between these two cases, notable differences appear in physical functioning, energy, and satisfaction levels. The isolated case has lower physical functioning and energy compared to the extended pelvic case, suggesting greater limitations in daily activities. Despite these physical constraints, the first case reports strong social functioning and moderate satisfaction, reflecting good emotional resilience and social support, though frequent experiences of pity from others may impact overall satisfaction. In contrast, the second case experiences slightly better emotional wellbeing and fewer perceived social barriers, and expresses higher satisfaction across life domains, likely due to improved physical capacity and energy.

When compared with general endometriosis populations (56), both patients’ scores in physical, social, and emotional functioning are notably higher than reported medians. This difference may stem from the isolated nature of the sciatic nerve lesion, as opposed to the more extensive intrapelvic endometriosis often seen in other cases, which tends to more broadly reduce overall QOL. The isolated localization likely limits some of the systemic effects common in widespread endometriosis, preserving certain aspects of QOL.

### 4.6 Peripheral nerve angiomatoid fibrous histiocytoma

AFHs are rare and low-grade soft-tissue lesions that typically arise from subcutaneous and deep dermal tissue of extremities. In rare cases, recurrence or tumor metastasis was noted (57). Based on our knowledge, there are no reports on AFH affecting a peripheral nerve and QOL following its surgical management. In our case, radical nerve resection with surrounding tissue debridement did not alter the patient’s postoperative functionality. However, because of the recurrence of the tumor and the need for more radical tissue debridement, the 1-year QOL was significantly reduced. The low QOL scores across multiple domains demonstrate the profound impact postoperative sequelae have on QOL despite the fact that the lesion is benign.

## 5 Study limitations

The limitations of this study include several key factors. First, the small sample size of only eight cases limits the generalizability of the findings, as rare peripheral nerve lesions have inherently low prevalence, making it difficult to draw broad conclusions applicable to a wider population. The retrospective nature of the study relies on existing medical records, which may lack detailed information on certain patient experiences or outcomes, potentially affecting the accuracy of the data. Furthermore, variations in lesion type, location, and individual patient characteristics introduce heterogeneity, which complicates comparisons and may influence QOL outcomes independently of the lesion type.

Another limitation is the lack of a control group, which makes it challenging to assess the relative impact of surgery on QOL compared to other treatment modalities. Since QOL assessments, including SF-36 and PNSQOL, were conducted only at specific intervals, they may not fully capture fluctuations in the patients’ QOL over time, particularly between the immediate postoperative period and the long-term follow-up.

Finally, the study’s reliance on self-reported measures of satisfaction and QOL may introduce bias, as patients’ subjective experiences can be influenced by factors beyond the clinical outcomes, such as support systems or personal expectations. These limitations highlight the need for future studies with larger sample sizes, prospective designs, and more standardized follow-up intervals to validate and expand upon the findings in this study.

## 6 Summary

This study presents long-term outcomes and QOL for eight patients who underwent surgical treatment for rare peripheral nerve lesions, highlighting the diversity in clinical presentation and the complexities of treatment. These lesions, including both primary and secondary origins, as well as benign and malignant types, underscore the challenges in diagnosis and management due to their rarity and varied characteristics. Surgical intervention was often complicated by adherence to surrounding structures, as seen in cases of NTRK-RSCN and endometriosis, leading to postoperative complications.

**Figure 9:**
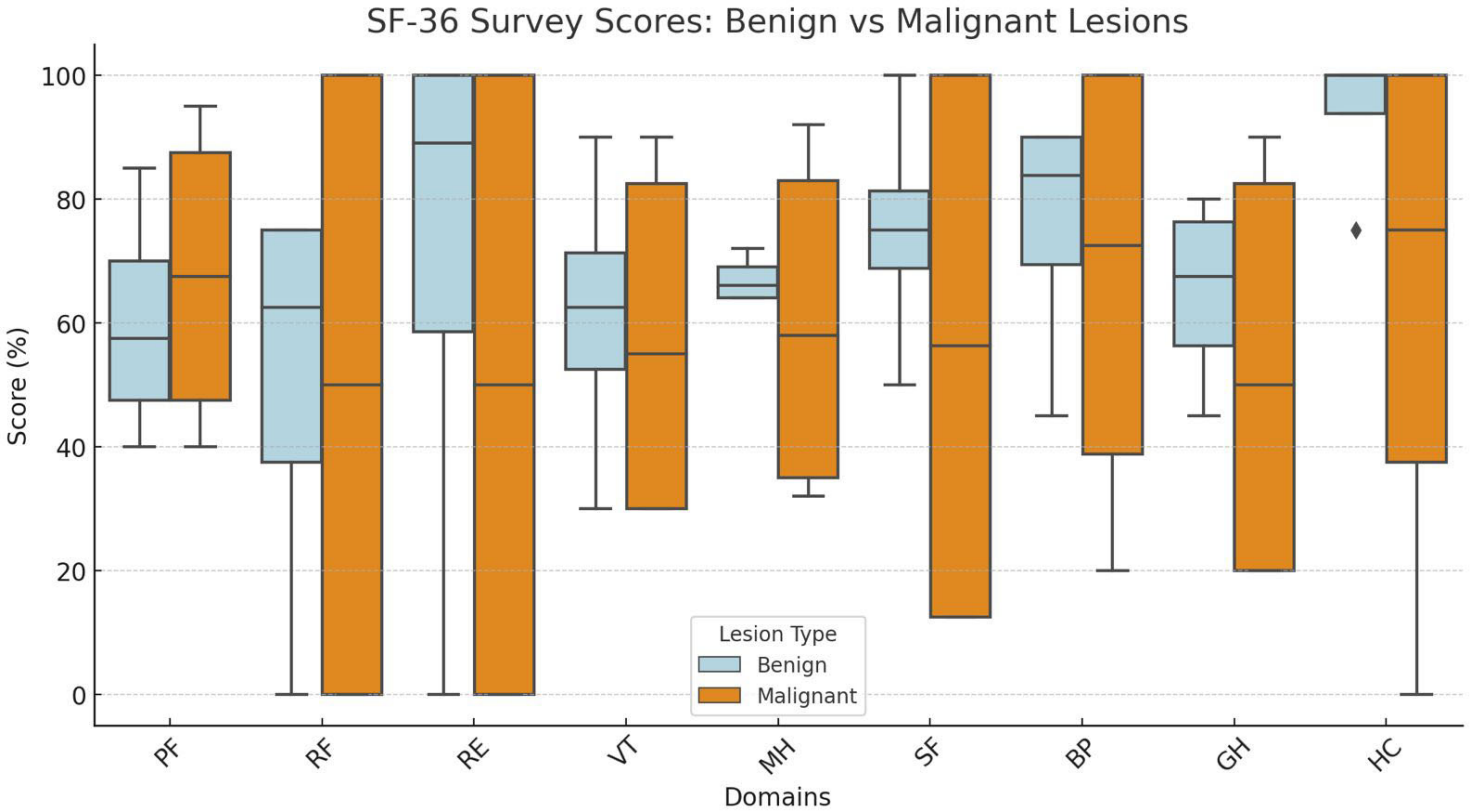
SF-36 scores with reference to the presence of malignancy: PF, physical functioning; RF, role limitations due to physical health; RE, role limitations due to emotional problems; VT, vitality (energy); MH, mental health (emotional well-being); SF, social functioning; BP, body pain; GH, general health; HC, health change.

Although benign lesions generally showed better overall QOL (**Figure 9**), this was more closely related to the level of nerve invasion and postoperative sequelae, such as pain or motor deficits, rather than the benign or malignant nature of the lesion itself. For instance, the benign saphenous nerve AFH case resulted in very low QOL due to significant postoperative complications, while the malignant tibial nerve NTRK-RSCN and brachial plexus lymphoma case had a high QOL, as there were minimal functional deficits and effective pain management. This suggests that postoperative outcomes, particularly regarding nerve function preservation and pain control, play a critical role in determining QOL, often more so than the lesion’s benign or malignant classification.

Psychological support is of great importance in managing malignant lesions, as it significantly contributes to maintaining and improving QOL. Even when physical and functional outcomes are favorable, the emotional and psychological challenges associated with a malignant diagnosis can impact wellbeing. Providing comprehensive psychological support helps patients cope with fears of recurrence, treatment side effects, and social or professional limitations, ultimately enhancing their overall resilience and QOL.

In conclusion, this study emphasizes the importance of an individualized approach in managing rare peripheral nerve lesions. Long-term outcomes were rather associated with the severity of nerve invasion and persistent symptomatology, rather than the involvement of malignancy. Despite a lack of standardized protocols, early intervention, targeted treatment strategies, and psychosocial support contributed positively to functionality and QOL in most cases.

## Data Availability

The original contributions presented in the study are included in the article/**Supplementary Material**, further inquiries can be directed to the corresponding author/s.
